# Supplementary figures and images for: An exploratory assessment of a multidimensional healthcare and economic data on COVID-19 in Nigeria
Source: Data Brief. 2020 Oct 21;33:106424. doi: 10.1016/j.dib.2020.106424 (PMC7576378; doi:10.1016/j.dib.2020.106424)

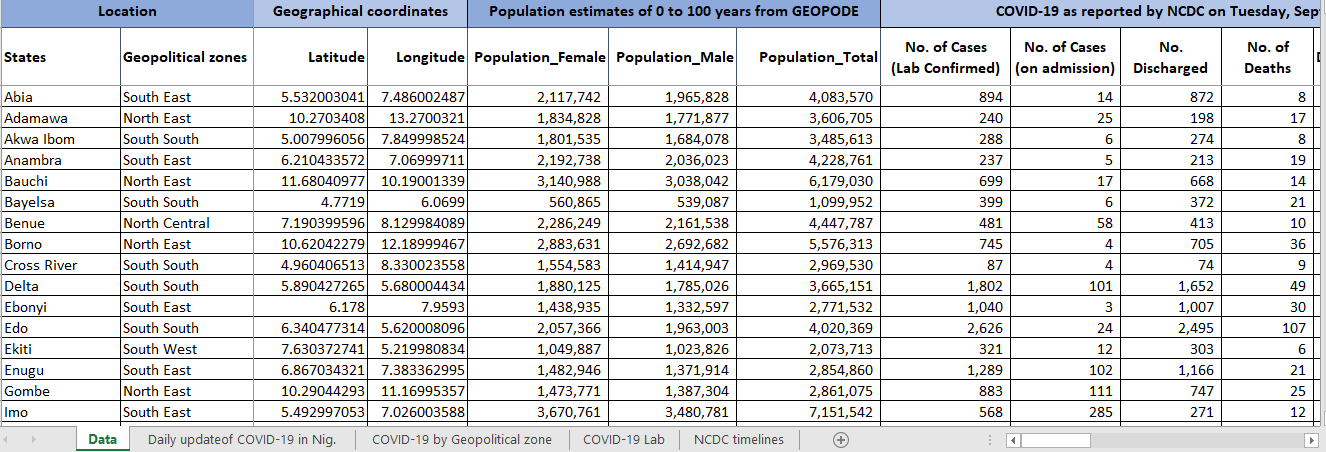

Supplement: Supplementary file 1 [file mmc1.zip › Figure 1.png]

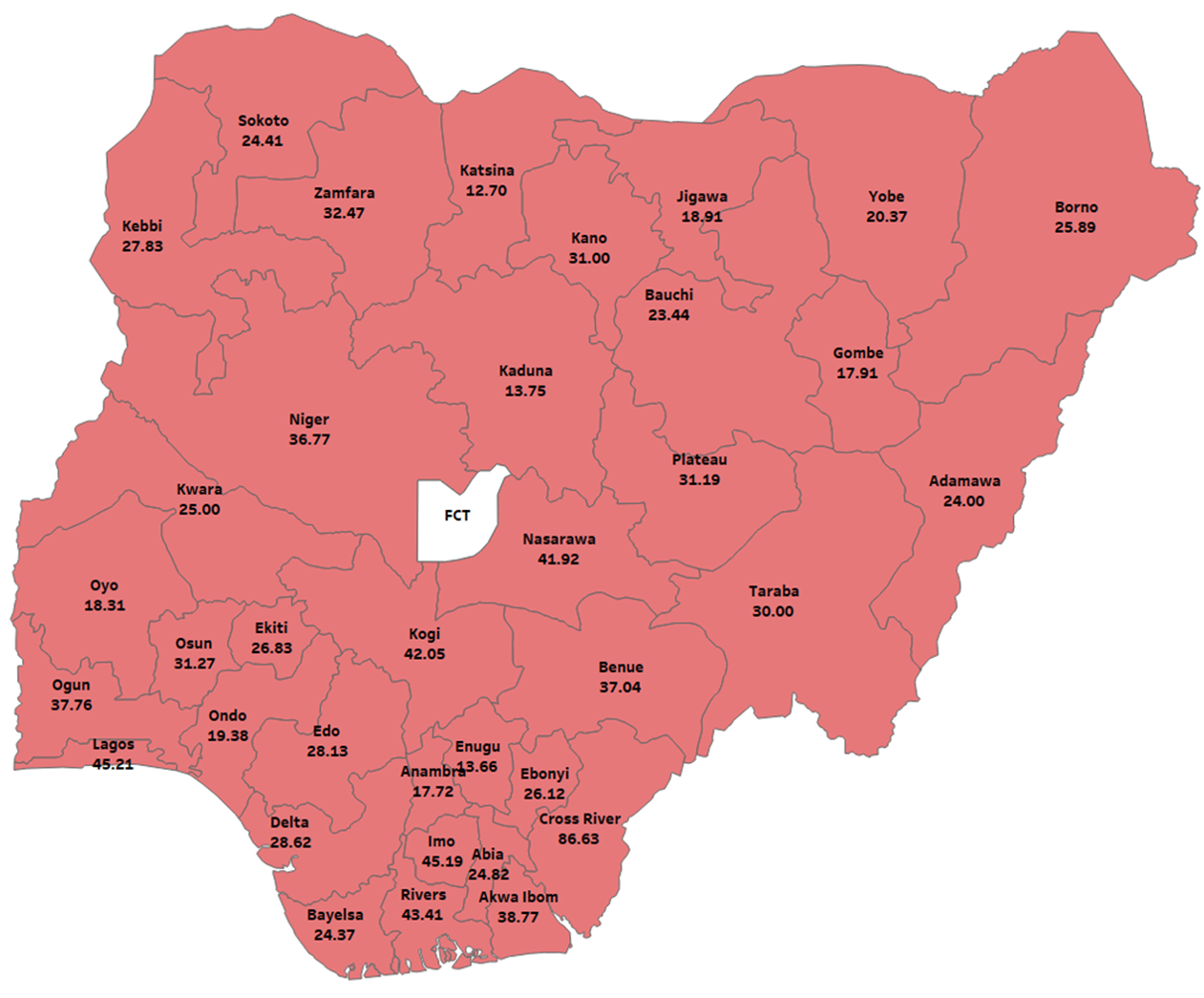

Supplement: Supplementary file 1 [file mmc1.zip › Figure 10.png]

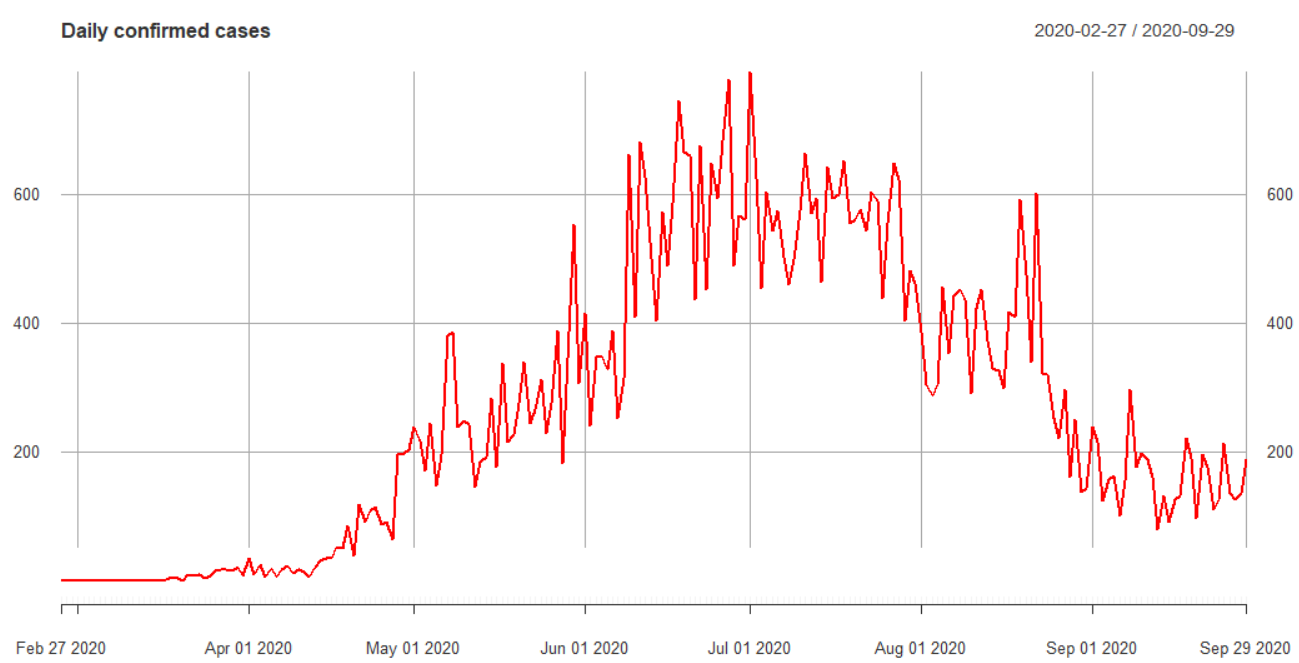

Supplement: Supplementary file 1 [file mmc1.zip › Figure 11.png]

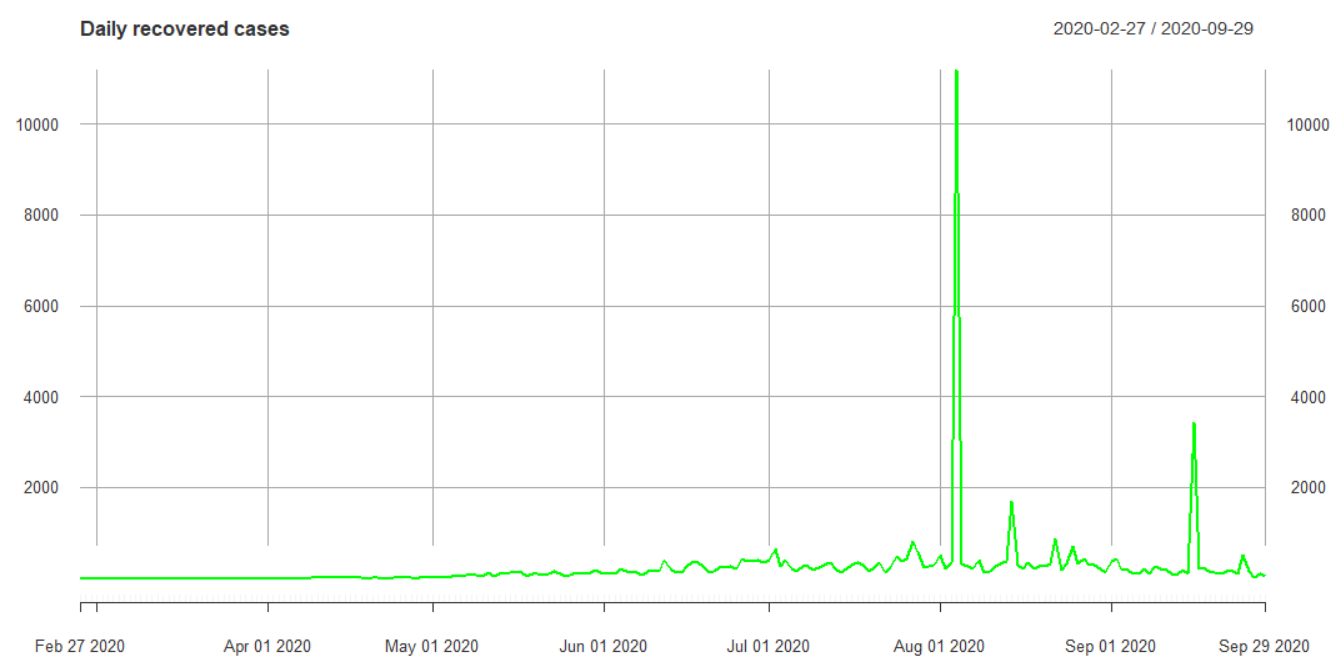

Supplement: Supplementary file 1 [file mmc1.zip › Figure 12.png]

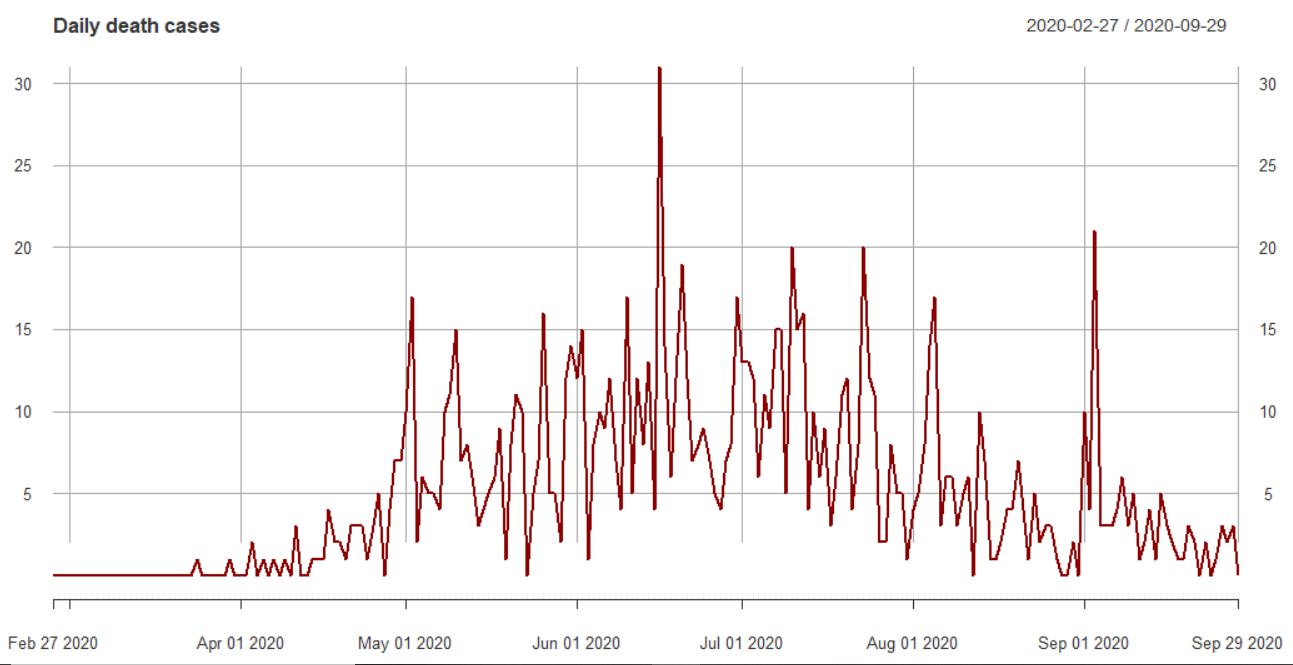

Supplement: Supplementary file 1 [file mmc1.zip › Figure 13.png]

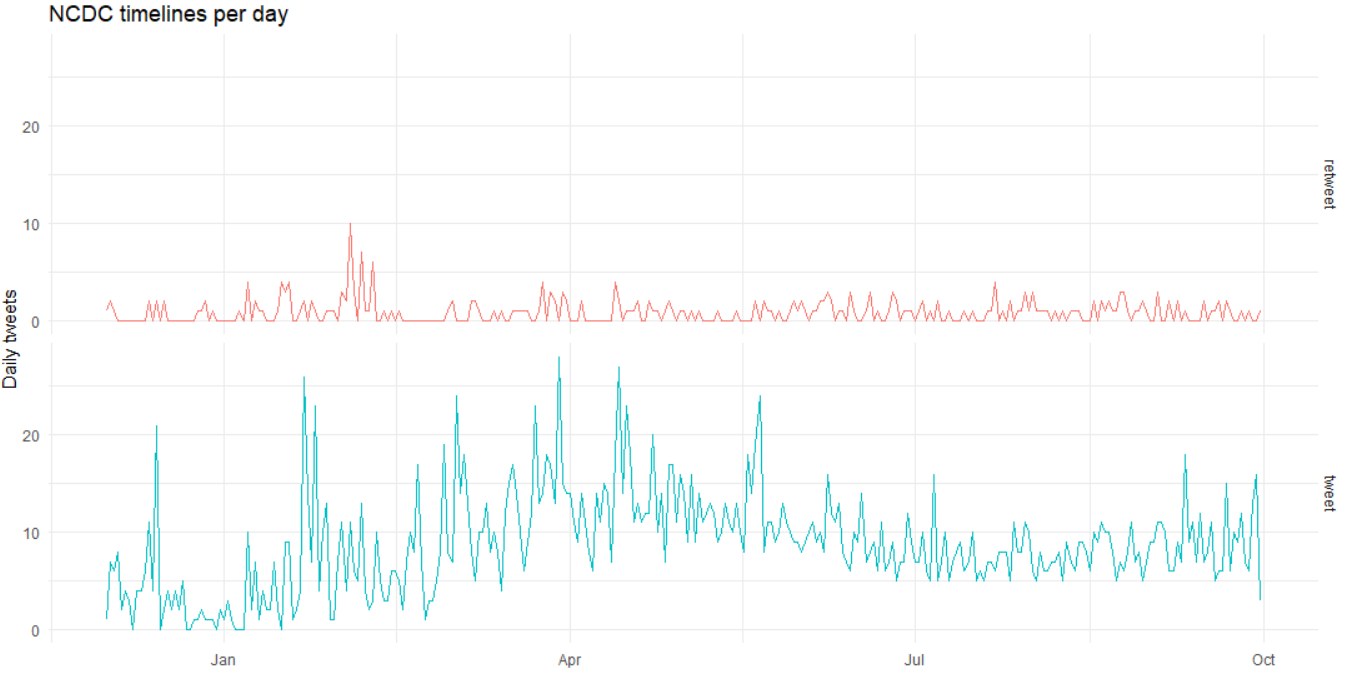

Supplement: Supplementary file 1 [file mmc1.zip › Figure 14.png]

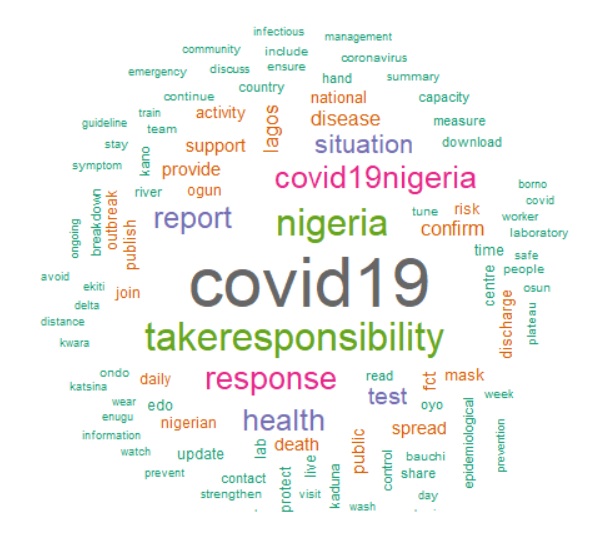

Supplement: Supplementary file 1 [file mmc1.zip › Figure 15.png]

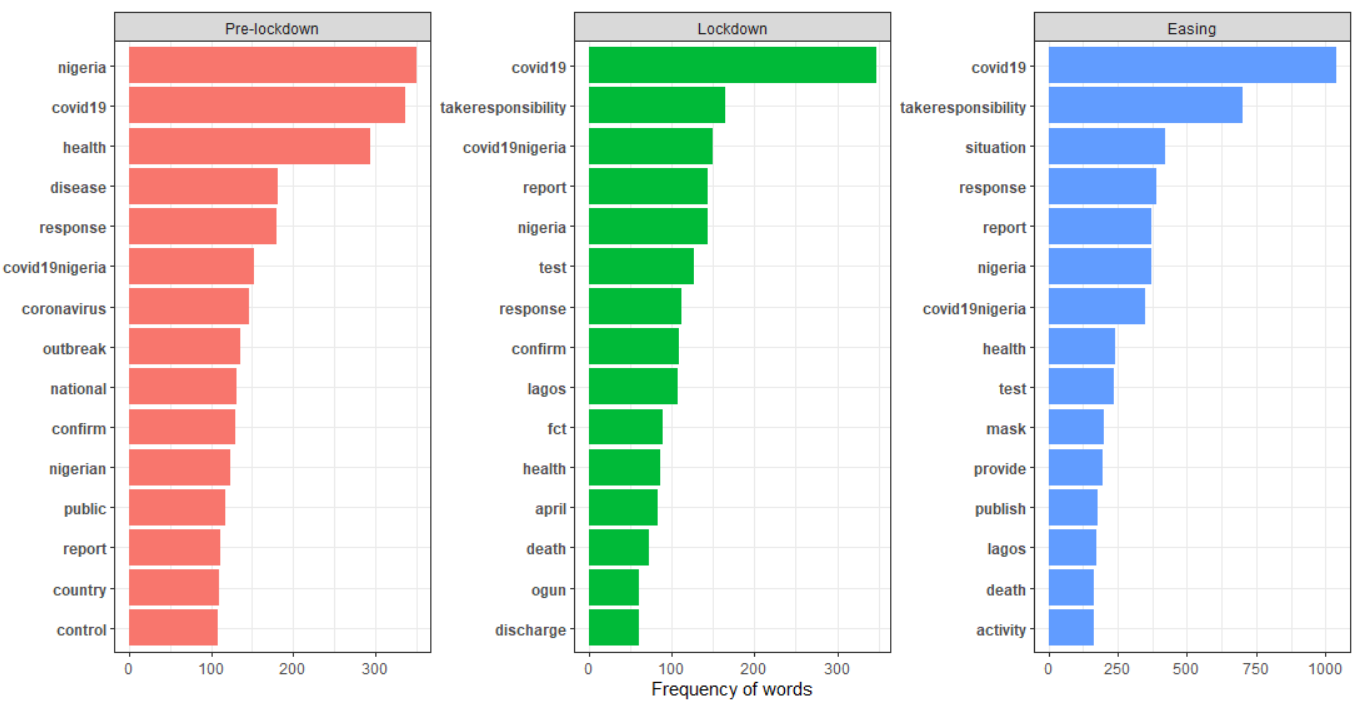

Supplement: Supplementary file 1 [file mmc1.zip › Figure 16.png]

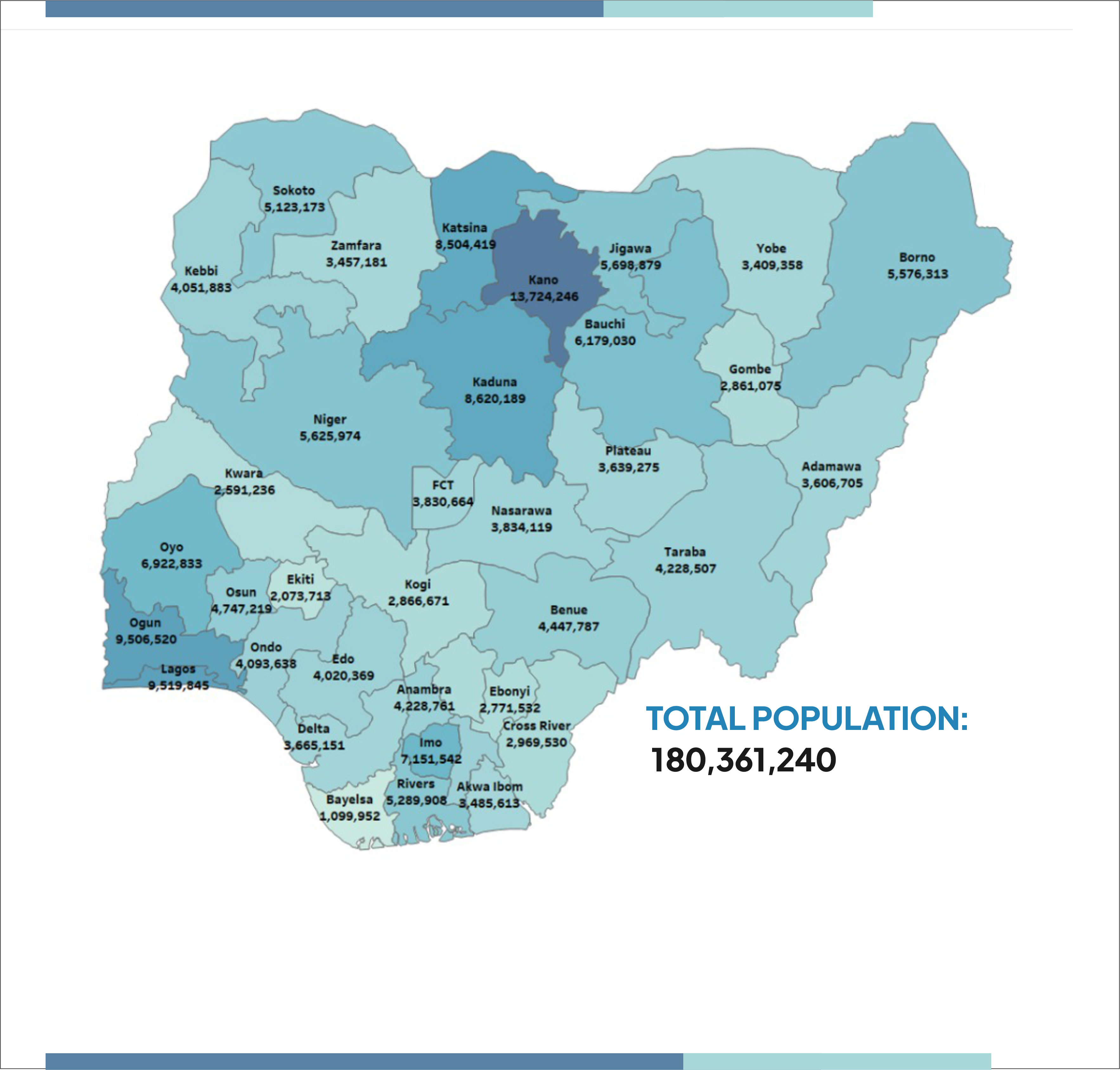

Supplement: Supplementary file 1 [file mmc1.zip › Figure 2.png]

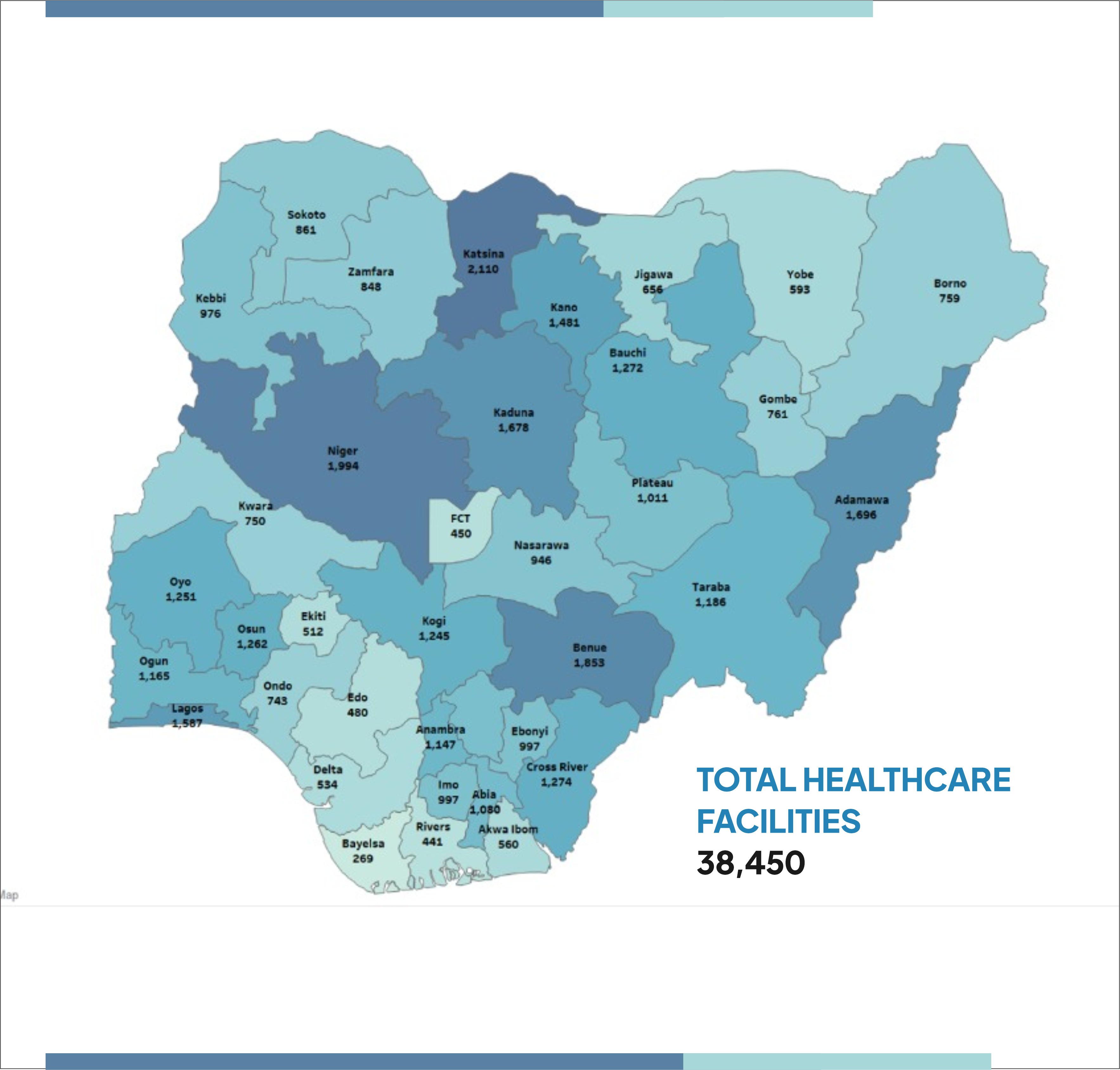

Supplement: Supplementary file 1 [file mmc1.zip › Figure 3.png]

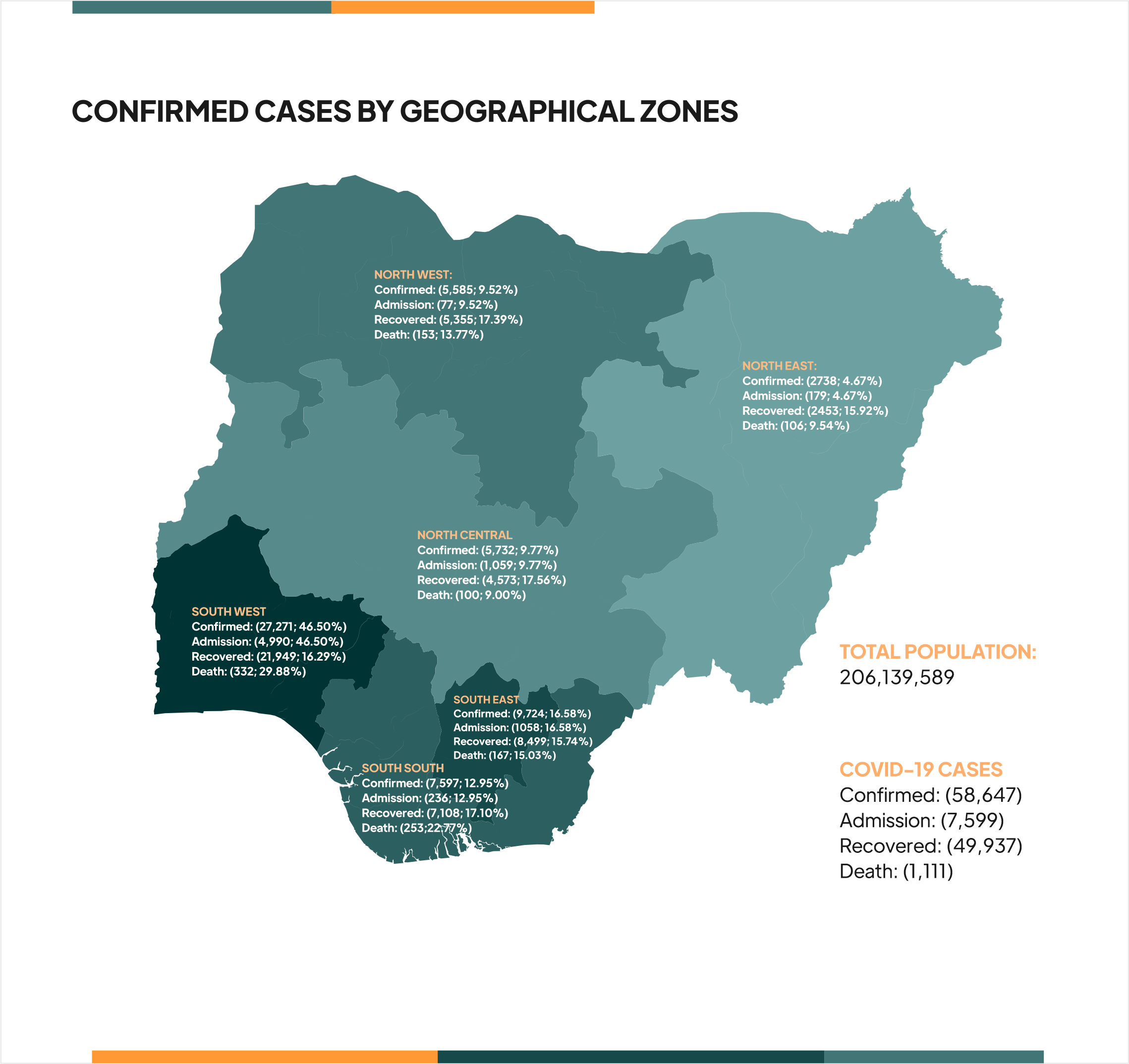

Supplement: Supplementary file 1 [file mmc1.zip › Figure 4.png]

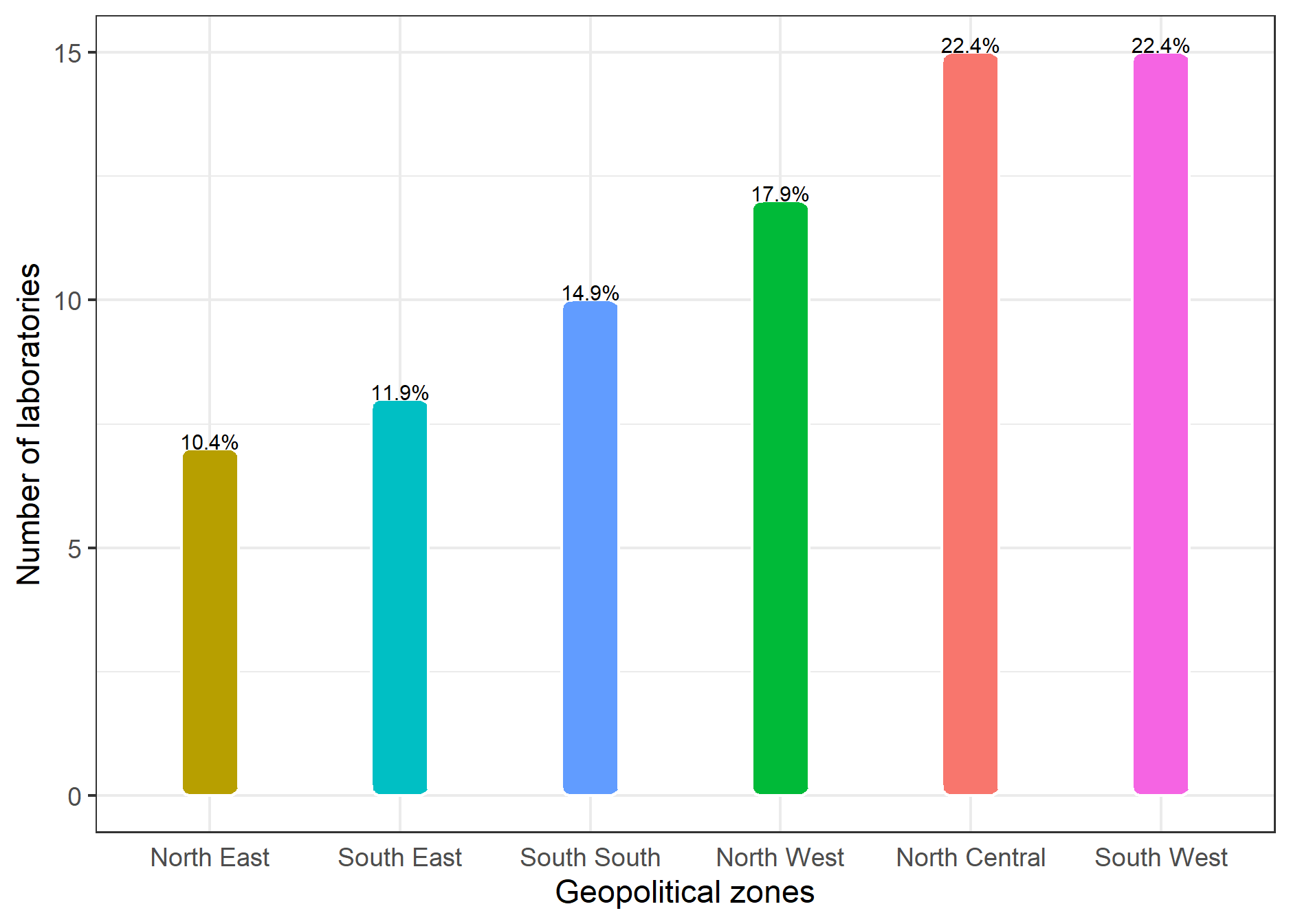

Supplement: Supplementary file 1 [file mmc1.zip › Figure 5.png]

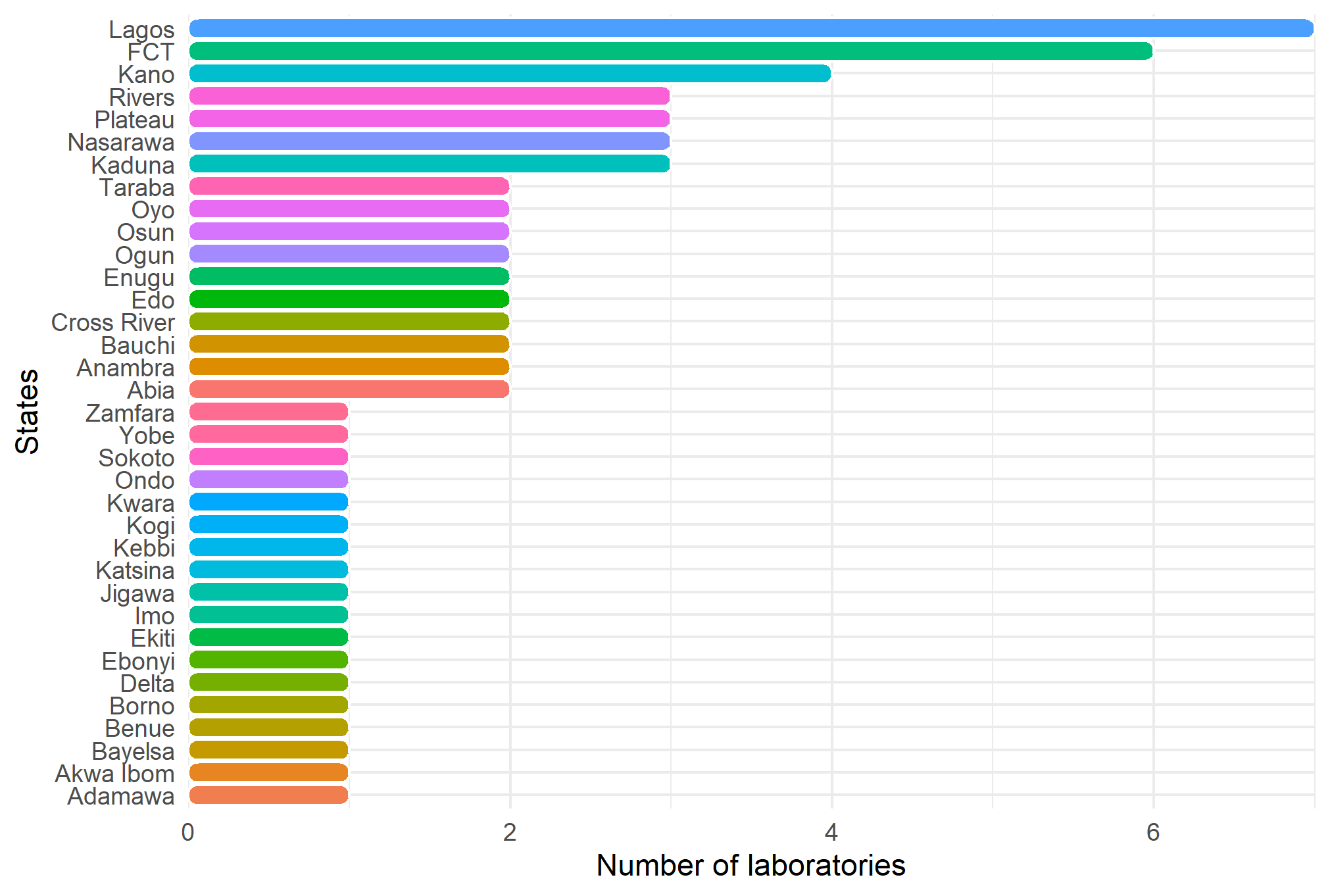

Supplement: Supplementary file 1 [file mmc1.zip › Figure 6.png]

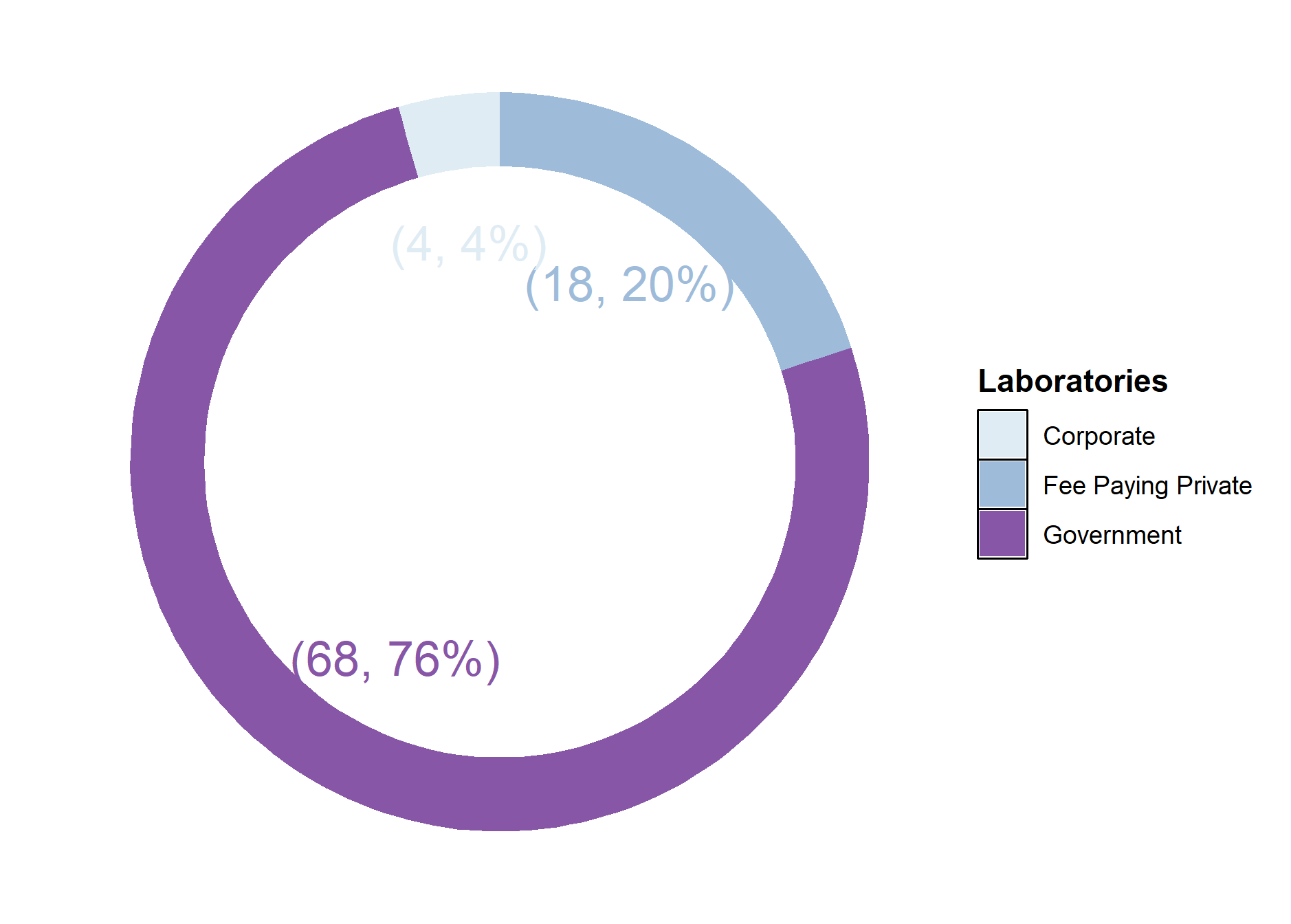

Supplement: Supplementary file 1 [file mmc1.zip › Figure 7.png]

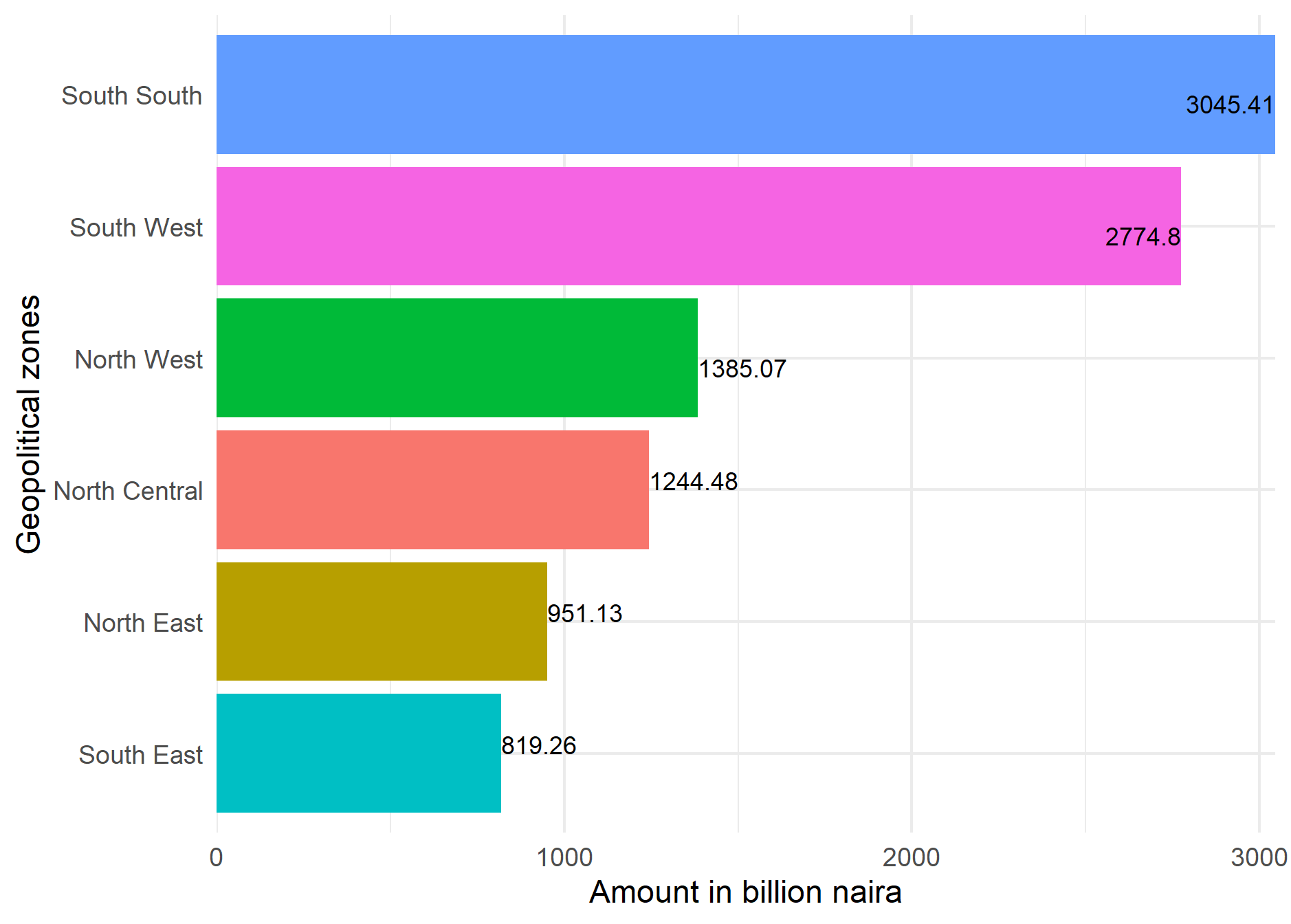

Supplement: Supplementary file 1 [file mmc1.zip › Figure 8.png]

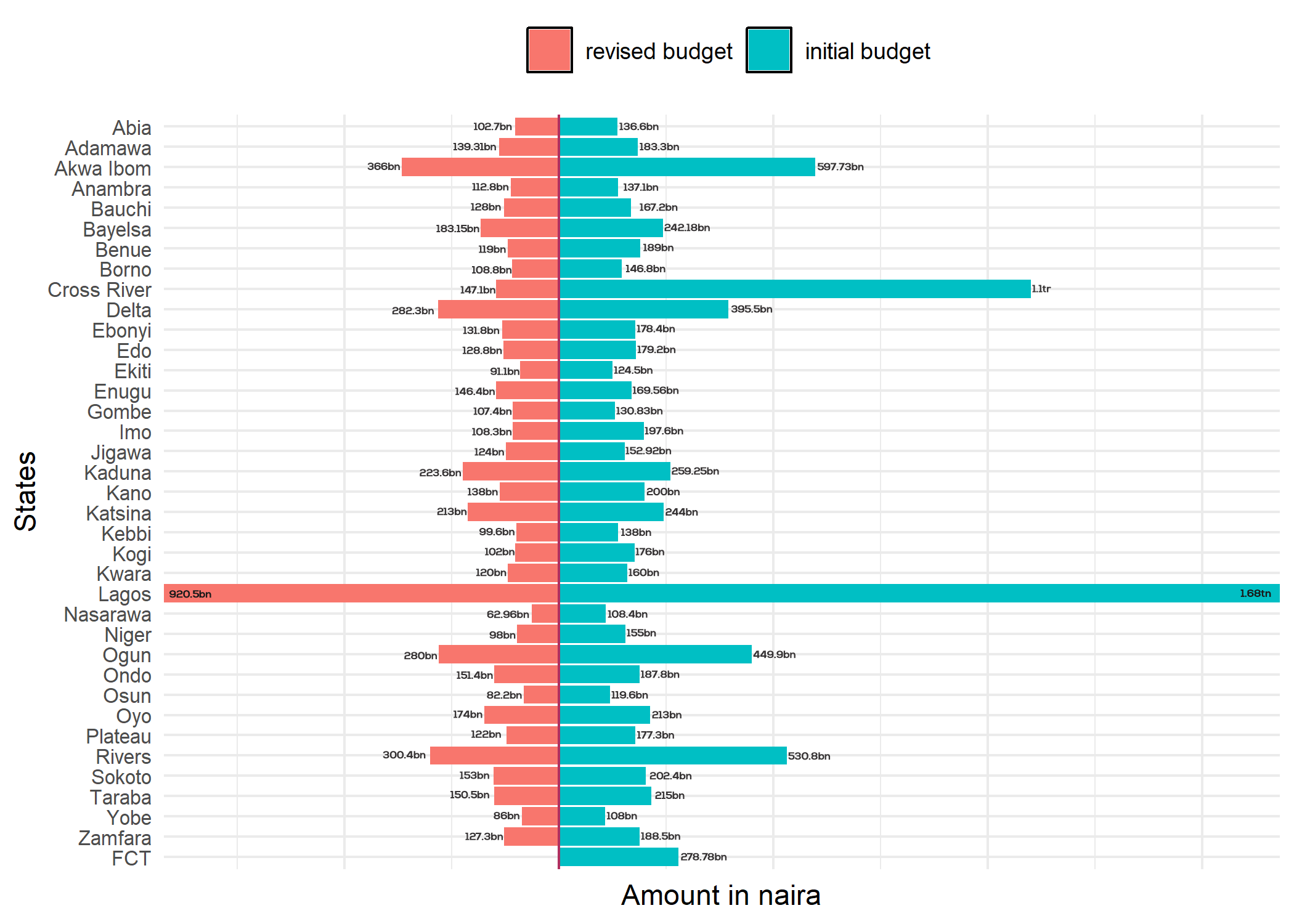

Supplement: Supplementary file 1 [file mmc1.zip › Figure 9.png]
